# Striatal small RNA remodeling in MPTP-induced Parkinson’s disease highlights coordinated downregulation of mitochondrial tsRNAs

**DOI:** 10.3389/fnagi.2026.1884207

**Published:** 2026-08-24

**Authors:** Mingyu Mao, Yan Wang, Qing Deng, Jun Xiang, Wentao Li

**Affiliations:** 1 Department of Neurology, Shanghai Municipal Hospital of Traditional Chinese Medicine, Shanghai University of Traditional Chinese Medicine, Shanghai, China; 2 Clinical College of Chinese Medicine, Gansu University of Chinese Medicine, Lanzhou, China; 3 Department of Epidemiology, Center for Global Health, School of Public Health, Nanjing Medical University, Nanjing, China

**Keywords:** mitochondrial dysfunction, mitochondrial tsRNAs, MPTP, pANDORA-seq, Parkinson’s disease, striatum

## Abstract

Parkinson’s disease (PD) is characterized by progressive nigrostriatal degeneration, yet the contribution of modification-rich small RNAs to PD pathology remains unclear. Here, we used PANDORA-seq to profile the striatal small RNA landscape in a subacute MPTP-induced mouse model of PD. MPTP-treated mice exhibited significant motor deficits together with reduced striatal tyrosine hydroxylase and dopamine transporter expression, confirming successful model establishment. Small RNA profiling revealed that transfer RNA-derived small RNAs and ribosomal RNA-derived small RNAs, rather than microRNAs, dominated the striatal small RNA transcriptome. Among the dysregulated small RNA classes, mitochondrial tsRNAs showed the most prominent and coordinated downregulation. Bioinformatic analysis suggested that predicted targets of differentially expressed mt-tsRNAs were enriched in synaptic organization, presynaptic function, membrane contact sites, and lipid-related pathways. In SH-SY5Y cells, transfection of an mt-tsRNA mimic partially reversed MPP^+^ induced increases in reactive oxygen species and restored mitochondrial membrane potential. These findings provide a modification-aware small RNA landscape of the PD striatum and identify mt-tsRNA downregulation as a notable feature of MPTP-induced parkinsonism.

## Introduction

Parkinson’s disease is a common progressive neurodegenerative disorder characterized pathologically by the progressive loss of dopaminergic neurons in the substantia nigra pars compacta (SNc), leading to dopamine depletion in the striatum and the emergence of characteristic motor symptoms (Soni et al., 2024). The pathogenesis of PD is currently considered multifactorial and involves several interconnected mechanisms, including α-synuclein aggregation, mitochondrial dysfunction, lysosomal/autophagic impairment, and neuroinflammation (Morris et al., 2024). However, the precise molecular basis underlying disease initiation and progression remains incompletely understood.

Epidemiological studies have suggested that pesticide exposure is associated with an increased risk of PD (Moisan et al., 2011; Steenland et al., 2013). Among environmental neurotoxins, 1-methyl-4-phenyl-1,2,3,6- tetrahydropyridine (MPTP) is one of the most widely used agents for modeling PD-related neurodegeneration. At sufficient doses, MPTP selectively damages the nigrostriatal dopaminergic system and induces dopaminergic neuronal loss, making it a well-established tool for generating PD mouse models (Vyas et al., 1986). Owing to its high lipophilicity, MPTP readily crosses the blood–brain barrier after systemic administration. Its active metabolite, 1-methyl-4-phenylpyridinium (MPP^+^), inhibits mitochondrial complex I (MCI) activity, thereby reducing ATP production, enhancing oxidative stress, disrupting mitochondrial ultrastructure and morphology, and ultimately promoting neuronal death (Mizuno et al., 1987). Notably, impaired MCI function in nigral dopaminergic neurons has been recognized as an important pathological feature of PD, and experimental disruption of MCI itself is sufficient to induce progressive parkinsonian neurodegeneration (González-Rodríguez et al., 2021). On this basis, the MPTP-induced mouse model provides a useful experimental system for investigating PD-related neuropathological processes, particularly those associated with mitochondrial injury and nigrostriatal dysfunction.

Small non-coding RNAs (sncRNAs) are a heterogeneous class of non-protein-coding RNAs, generally shorter than 200 nucleotides, that include microRNAs (miRNAs), small interfering RNAs (siRNAs), PIWI-interacting RNAs (piRNAs), tRNA-derived small RNAs (tsRNAs), rRNA-derived small RNAs (rsRNAs), small nucleolar RNAs (snoRNAs), and small nuclear RNAs (snRNAs) (Gou et al., 2023; Xiong and Zhang, 2023). These molecules participate in a broad range of biological processes through post-transcriptional and transcriptional regulation of gene expression (Knittel et al., 2025; Sarkies, 2024; Welsh and Gardini, 2023), and have also been implicated in the regulation of cell death, development, immunity, and inflammatory responses (Chen et al., 2025; Muraoka et al., 2024; Wu et al., 2025; Yu et al., 2025). Increasing evidence indicates that sncRNAs are involved in the pathogenesis of neurological disorders, including Alzheimer’s disease (AD) and PD. In AD, aberrant sncRNA expression has been identified in both central and peripheral samples, and these RNAs have been linked to multiple pathological processes, including gene regulatory dysfunction, amyloid-β production, tau phosphorylation, inflammation, synaptic plasticity, and neuronal survival (Lauretti et al., 2021). In PD, miRNAs have been extensively studied as potential biomarkers and are considered among the most promising non-coding RNA candidates for translational application and therapeutic targeting (Kuo et al., 2021).

Despite these advances, the small RNA landscape in PD, particularly in disease-relevant brain regions such as the striatum, remains incompletely characterized. A major technical limitation of conventional small RNA sequencing is its reduced ability to detect RNA species carrying terminal chemical modifications or internal base modifications, which can interfere with adaptor ligation and reverse transcription. As a result, specific subclasses of sncRNAs – especially tsRNAs and rsRNAs – may be underrepresented or overlooked, despite growing evidence that they may have important regulatory functions. To address this limitation, PANDORA-seq (panoramic RNA display by overcoming RNA modification aborted sequencing) was developed to reduce sequencing bias caused by RNA end chemistry and internal RNA modifications (Shi et al., 2021). Compared with conventional small RNA sequencing, PANDORA-seq more effectively captures previously masked populations of modified sncRNAs, particularly tsRNAs and rsRNAs, thereby enabling a more comprehensive and accurate characterization of the small RNA transcriptome.

In the present study, we applied PANDORA-seq to profile small RNAs in the striatum of control and MPTP-induced PD model mice. By integrating differential expression and bioinformatic enrichment analyses, we aimed to systematically characterize the striatal sncRNA landscape under PD-like conditions and to identify candidate small RNA regulatory networks that may contribute to disease-related molecular pathology. In addition, we further validated the effects of the lead RNA on mitochondrial function, providing experimental support for its potential involvement in PD-related mitochondrial dysregulation.

## Results

### Establishment and validation of the subacute MPTP-induced PD mouse model

To establish a PD-like mouse model, C57BL/6J mice were intraperitoneally injected with MPTP for 10 consecutive days. During the modeling period, mice in the MPTP group gradually exhibited parkinsonian-like manifestations, including transient tremor, hindlimb rigidity, kyphosis, and reduced spontaneous activity, whereas WT mice showed no obvious abnormal behaviors.

**FIGURE 1:**
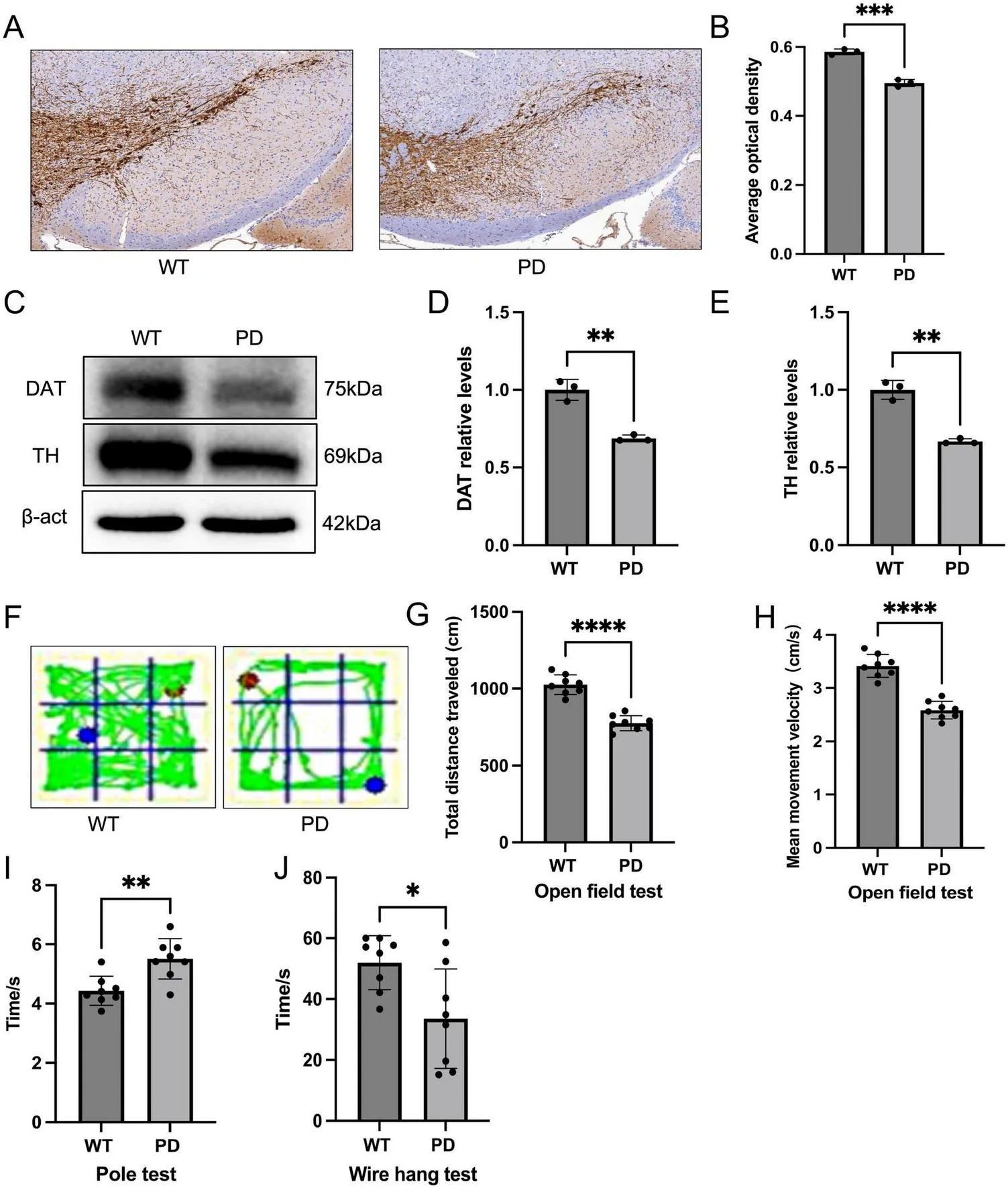
**(A)** Representative images of TH immunohistochemical staining in the substantia nigra of WT and PD mice. **(B)** Quantification of TH immunoreactivity by AOD, showing a significant reduction in the PD group compared with the WT group. **(C)** Representative western blot images of DAT and TH protein expression in the striatum of WT and PD mice, with β-actin used as the loading control. **(D, E)** Quantitative analysis of DAT and TH protein levels, showing significant decreases in PD mice compared with WT controls. **(F)** Representative locomotor trajectories of WT and PD mice in the open-field test. **(G)** Quantification of total distance traveled in the open-field test. **(H)** Quantification of mean movement velocity in the open-field test. **(I)** Pole test performance, presented as the total time required to complete the task. **(J)** Wire hang test performance, presented as latency to fall (*P<0.05, **P<0.01, ***P<0.001, ****P<0.0001 versus WT).

Behavioral assessments performed within 48 h after the final injection revealed significant motor deficits in MPTP-treated mice. In the open-field test, representative movement trajectories showed a marked reduction in exploratory behavior in PD mice compared with WT mice (Figure 1F). Quantitative analysis demonstrated that both the total distance traveled and the mean movement velocity were significantly decreased in the PD group (Figures 1G, H), indicating impaired spontaneous locomotor activity. In the pole test, the total time required to complete the task was significantly prolonged in PD mice relative to WT controls (Figure 1I), suggesting impaired motor coordination and bradykinesia. In the wire hang test, PD mice displayed a significantly shorter latency to fall than WT mice (Figure 1J), indicating reduced muscle strength and motor endurance.

To further validate successful model establishment at the histological and molecular levels, dopaminergic neuronal markers in the striatum were examined. TH immunohistochemical staining showed an obvious reduction in TH-positive fibers in the substantia nigra of PD mice compared with WT mice (Figure 1A). Consistently, quantitative analysis of the average optical density (AOD) demonstrated a significant decrease in TH immunoreactivity in the PD group (Figure 1B). In addition, western blot analysis showed that the protein levels of dopamine transporter (DAT) and tyrosine hydroxylase (TH) were both significantly reduced in the striatum of PD mice compared with WT controls (Figures 1C–E). Taken together, these behavioral impairments, along with the loss of striatal dopaminergic markers detected by immunohistochemistry and western blotting, indicate that the subacute MPTP-induced PD mouse model was successfully established.

### Global sncRNA profiling of mouse brain by PANDORA-seq

**FIGURE 2:**
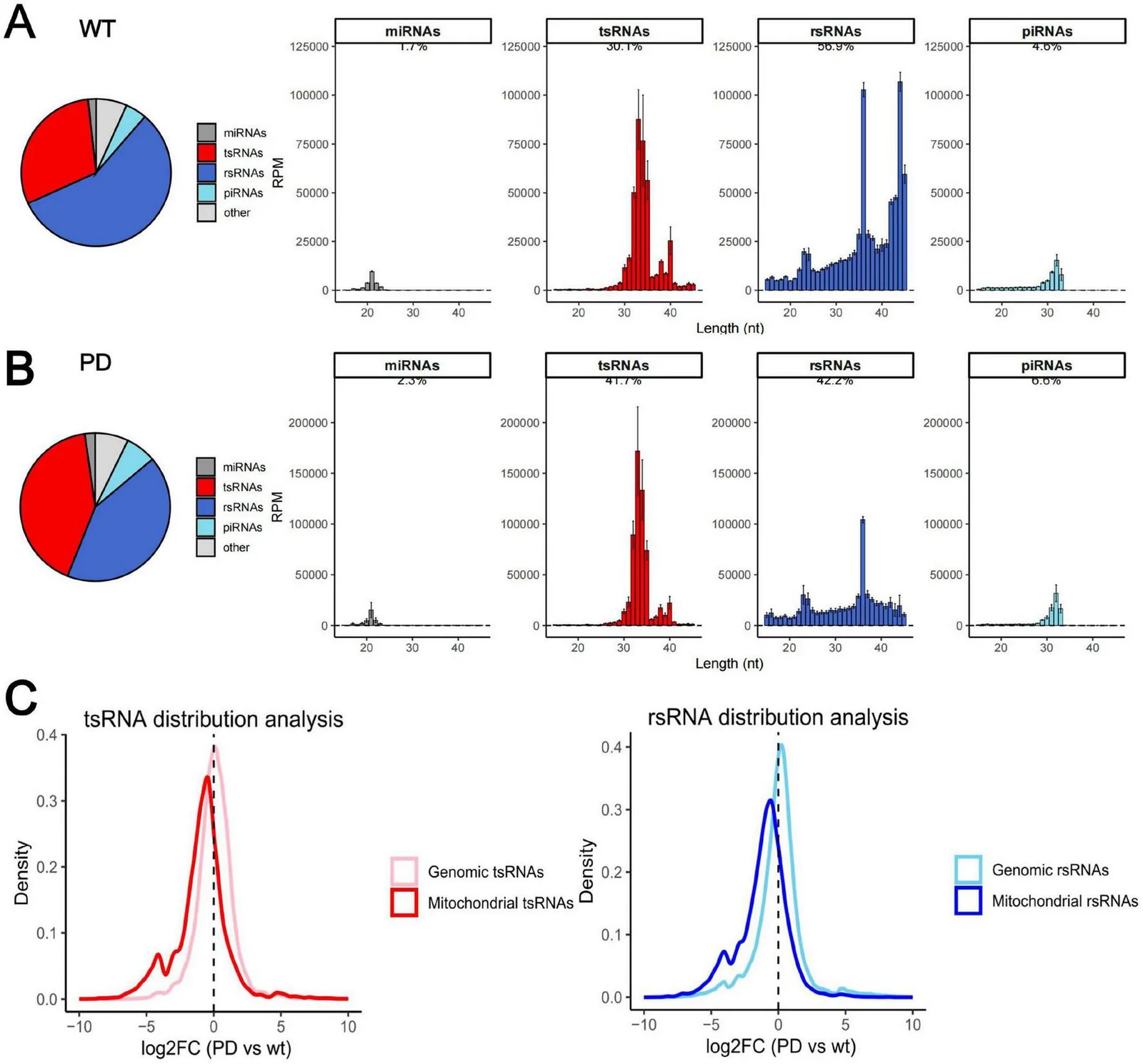
**(A)** Small RNA composition and length distribution profiles in WT mouse brains. **(B)** Small RNA composition and length distribution profiles in PD mouse brains. **(C)** Distribution analysis of genomic- and mitochondrial-derived tsRNAs and rsRNAs.

To characterize the small RNA landscape in WT and PD mouse brains, we performed PANDORA-seq and analyzed the small RNA composition of the two groups separately. The sequencing data have been deposited in the Genome Sequence Archive (GSA) under accession number: CRA045273. Public access link: https://ngdc.cncb.ac.cn/gsa/s/y8x0oG26. As shown in Figures 2A, B, the overall small RNA profiles of both WT and PD brains differed markedly from the canonical small RNA landscape typically dominated by miRNAs. In both groups, tsRNAs and rsRNAs represented the predominant small RNA biotypes, whereas miRNAs and piRNAs accounted for only a minor proportion of the annotated reads.

Specifically, in WT brains, rsRNAs and tsRNAs constituted the major fractions of total mapped reads, whereas miRNAs and piRNAs represented only a small proportion (Figure 2A). A similar overall composition was observed in PD brains, in which tsRNAs and rsRNAs also remained the dominant small RNA classes (Figure 2B). These findings indicate that tsRNAs and rsRNAs are major components of the brain small RNA transcriptome under both physiological and PD conditions.

Length distribution analysis further demonstrated distinct size preferences for different small RNA classes in both groups. In WT and PD samples, miRNAs were enriched around the expected ∼22 nt size range, tsRNAs were mainly distributed at approximately 30–35 nt, rsRNAs displayed a broader distribution with major peaks in the 30–40 nt range, and piRNAs were primarily enriched around 30–33 nt (Figures 2A, B). These results suggest that the major small RNA classes detected by PANDORA-seq exhibit conserved but distinguishable length characteristics in both WT and PD brains.

We next examined the distribution patterns of genomic- and mitochondrial-derived tsRNAs and rsRNAs. As shown in Figure 2C, both genomic tsRNAs and mitochondrial tsRNAs were detected in the brain small RNA pool, and their log2 fold change distributions showed broadly comparable patterns. Similarly, genomic rsRNAs and mitochondrial rsRNAs also exhibited overlapping distribution trends (Figure 2C). These results indicate that mitochondrial-derived tsRNAs and rsRNAs constitute a detectable component of the brain small RNA transcriptome and may contribute to the altered small RNA landscape in PD. Collectively, these data demonstrate that PANDORA-seq captures a brain small RNA profile enriched for tsRNAs and rsRNAs in both WT and PD mice, rather than one predominantly composed of miRNAs.

### PCoA revealed robust separation between WT and MT samples, with mt-tsRNAs showing the strongest differential expression pattern

**FIGURE 3:**
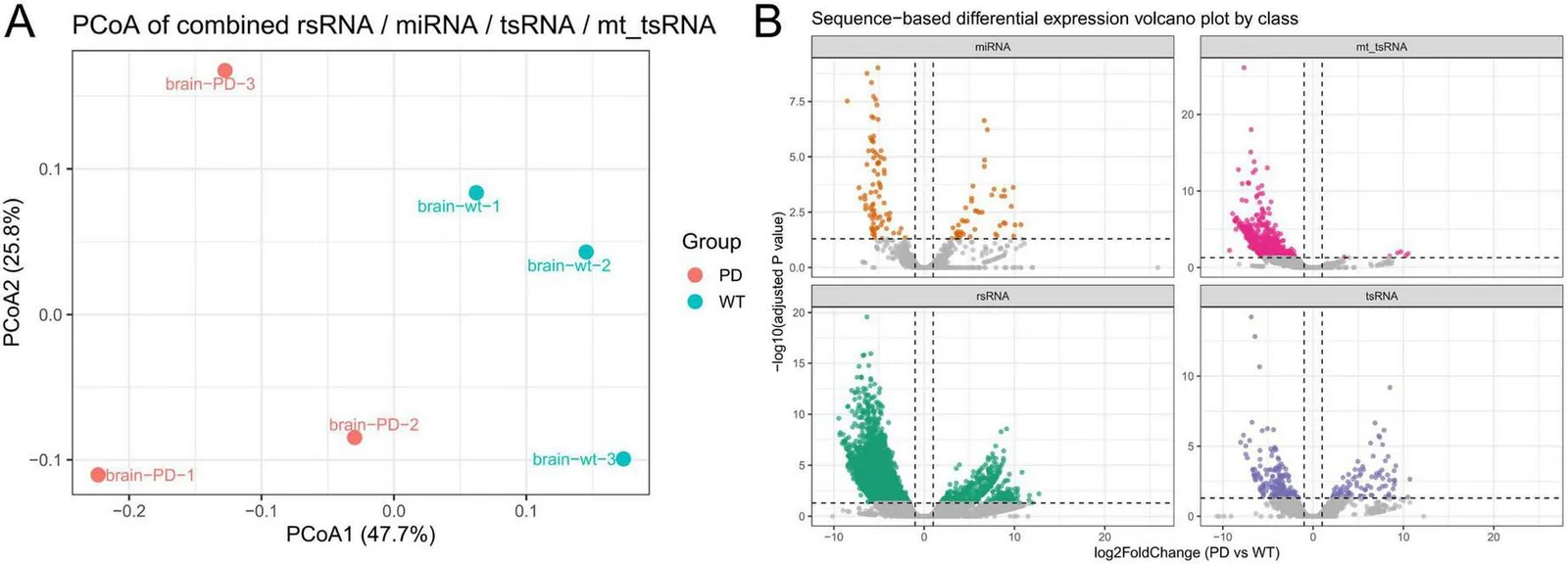
**(A)** PCoA of combined rsRNA, miRNA, tsRNA, and mt-tsRNA expression profiles in brain samples, showing clear separation between WT and PD groups (*n* = 3 per group). **(B)** Sequence-based differential expression volcano plots of miRNAs, mt-tsRNAs, rsRNAs, and tsRNAs in PD versus WT brains. The *x*-axis indicates log2 fold change (PD vs. WT), and the *y*-axis indicates -log10 adjusted *P* value. Vertical dashed lines represent fold-change thresholds, and the horizontal dashed line indicates the significance cutoff.

To assess the global effect of the mutation on small RNA expression, we performed principal coordinates analysis (PCoA) based on normalized expression data for rsRNAs, miRNAs, tsRNAs, and mt-tsRNAs. WT and MT samples were clearly separated, with PCoA1 explaining 47.7% of the total variance and PCoA2 explaining 25.8% (Figure 3A). Biological replicates within each group clustered relatively closely, indicating good reproducibility.

We next examined differential expression patterns across major sncRNA classes. Volcano plot analysis showed that miRNAs, rsRNAs, and genomic tsRNAs exhibited comparatively balanced distributions of up- and downregulated species (Figure 3B). In contrast, mt-tsRNAs displayed a marked skew toward negative log2FC values, indicating that a substantial fraction of mt-tsRNAs was reduced in MT relative to WT samples. These results suggest that mt-tsRNAs represent the sncRNA subclass most prominently altered in association with the mutation.

### Mt-tsRNA mimics transfection efficiency and functional assessment in MPP^+^ treated SH-SY5Y cells

**FIGURE 4:**
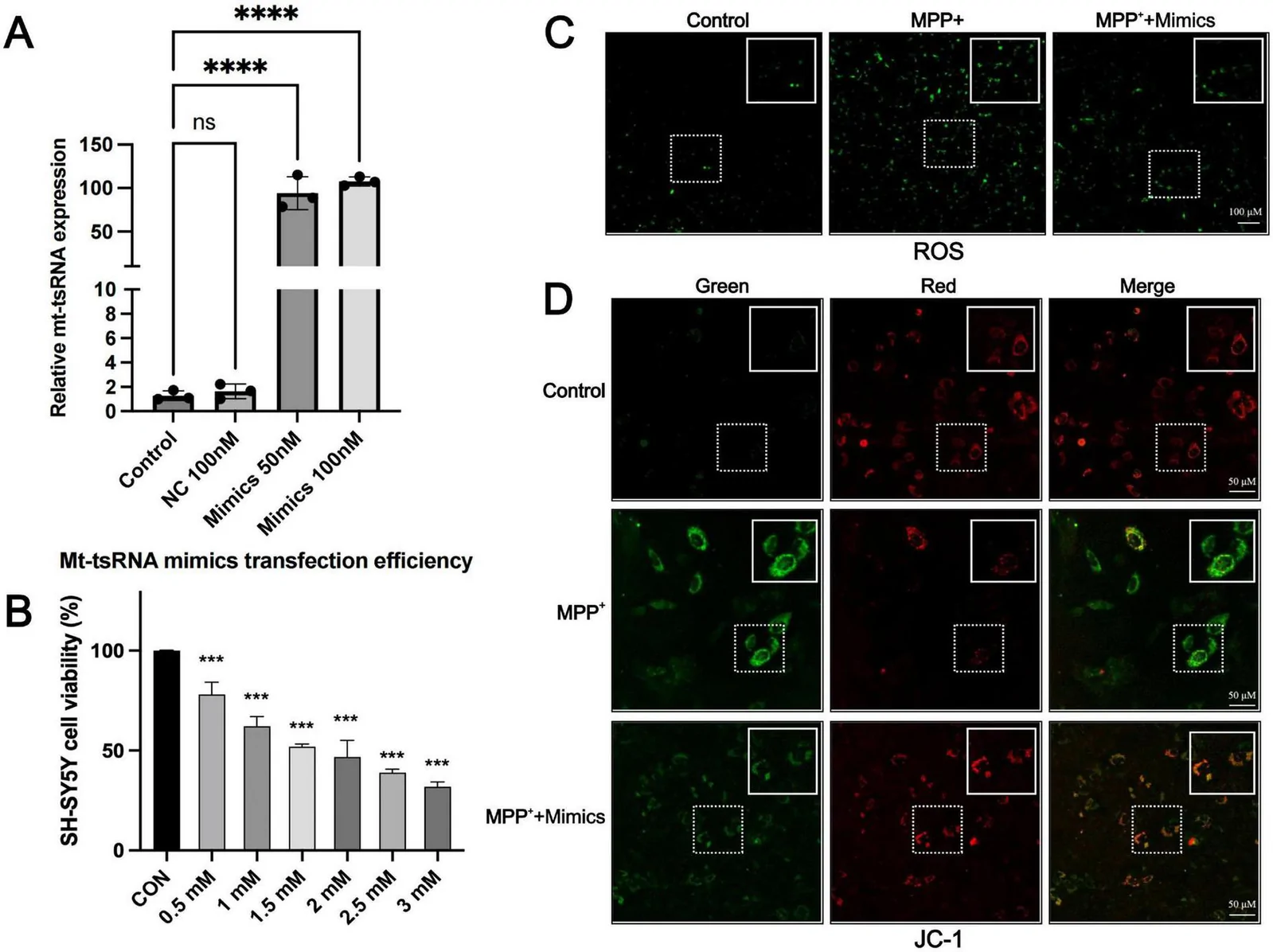
**(A)** RT-qPCR analysis of the target mt-tsRNA expression in SH-SY5Y cells after transfection with mt-tsRNA mimics at different concentrations. **(B)** CCK-8 assay showing the viability of SH-SY5Y cells treated with different concentrations of MPP^+^. **(C)** Representative confocal images of intracellular ROS staining in Control, MPP^+^, and MPP^+^ + Mimics groups. **(D)** Representative confocal images of JC-1 staining in Control, MPP^+^, and MPP^+^ + Mimics groups. (*ns*, not significant; ***P<0.001; ****P<0.0001).

We selected the most significantly dysregulated lead mt-tsRNA for further functional validation. To determine the optimal transfection condition for mt-tsRNA mimics, SH-SY5Y cells were transfected with different concentrations of mimics, and mt-tsRNA expression was measured by RT–qPCR. As shown in Figure 4A, no significant difference in mt-tsRNA expression was observed between the untreated control group and the negative control (NC) group, indicating that the transfection procedure itself did not affect endogenous mt-tsRNA levels. In contrast, transfection with mt-tsRNA mimics significantly increased mt-tsRNA expression, with both 50 nM and 100 nM yielding robust overexpression. 50 nM was selected for subsequent experiments.

To establish an *in vitro* PD-like injury model, SH-SY5Y cells were exposed to increasing concentrations of MPP^+^, and cell viability was evaluated using the CCK-8 assay. MPP^+^ treatment significantly reduced cell viability in a concentration-dependent manner (Figure 4B). Among the tested concentrations, 1.5 mM MPP^+^ produced a marked reduction in viability while preserving a sufficient proportion of viable cells for downstream functional assays, and was therefore chosen as the working concentration for subsequent experiments.

We next examined whether mt-tsRNA overexpression affected oxidative stress and mitochondrial function in MPP^+^ treated SH-SY5Y cells. Intracellular ROS levels were assessed by confocal microscopy. As shown in Figure 4C, ROS fluorescence was weak in control cells but was markedly increased following MPP^+^ exposure, indicating substantial oxidative stress. Notably, mt-tsRNA mimics transfection attenuated the MPP^+^ induced increase in ROS fluorescence, suggesting that mt-tsRNA overexpression partially alleviated oxidative stress.

Mitochondrial membrane potential was further evaluated by JC-1 staining. In control cells, JC-1 staining showed predominant red fluorescence, consistent with intact mitochondrial membrane potential. In contrast, MPP^+^ treatment led to a clear increase in green fluorescence accompanied by a reduction in red fluorescence, indicative of mitochondrial depolarization. Importantly, mt-tsRNA mimics transfection partially reversed this fluorescence shift in MPP^+^ treated cells (Figure 4D), suggesting an amelioration of mitochondrial membrane potential loss. Collectively, these findings indicate that mt-tsRNA overexpression exerts a protective effect against MPP^+^ induced oxidative stress and mitochondrial dysfunction in SH-SY5Y cells.

### GO enrichment results

**FIGURE 5:**
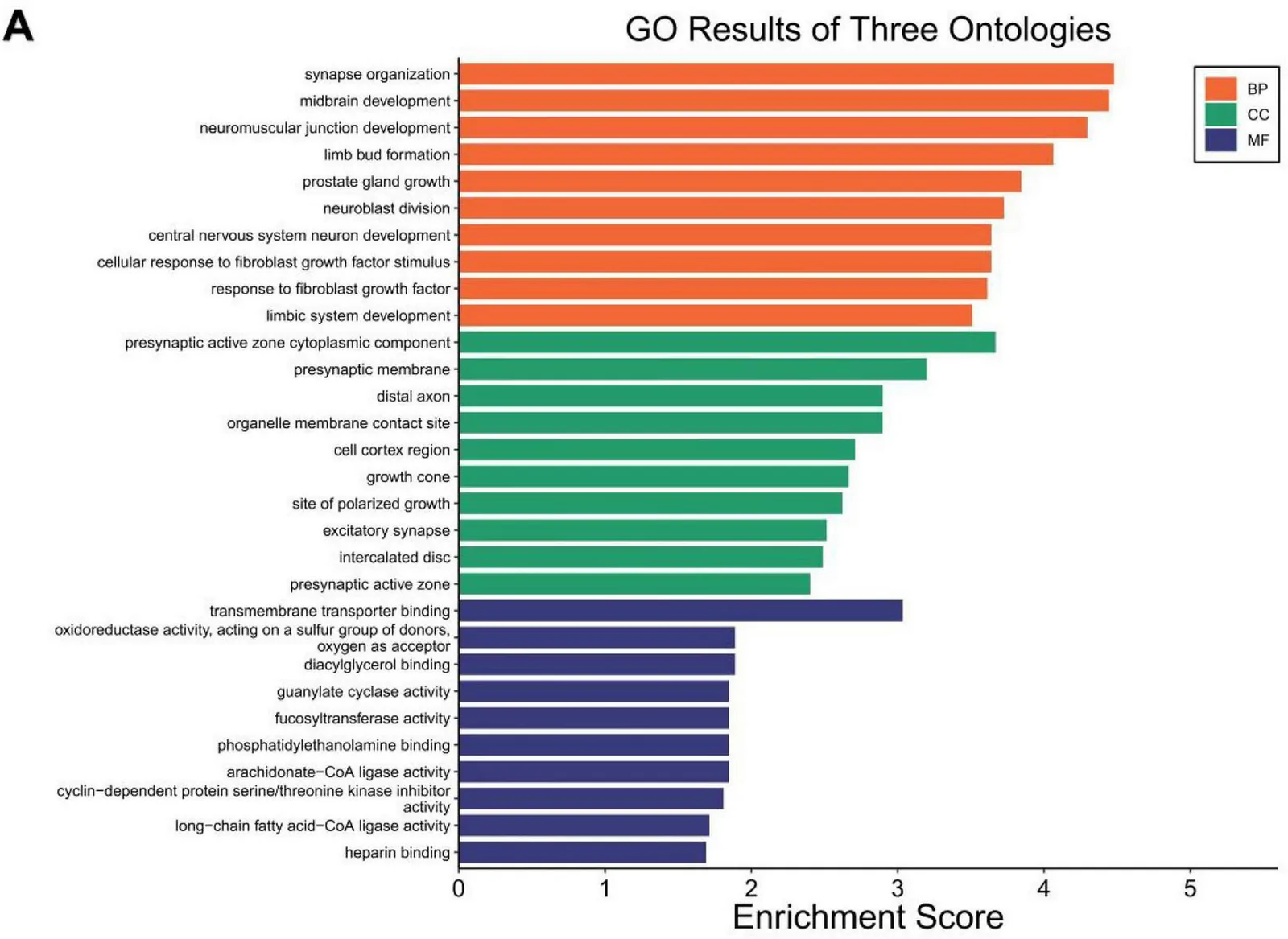
**(A)** Bar plot showing the top enriched GO terms from the three ontology categories, including BP, CC, and MF, ranked by enrichment score.

For miRDB target prediction, mt-tsRNAs ranging from 15 to 25 nt in length were screened (detailed in Supplementary Table 1). To systematically investigate the potential biological functions of the differentially expressed sncRNA target genes, Gene Ontology (GO) enrichment analysis was performed across the Biological Process (BP), Cellular Component (CC), and Molecular Function (MF) categories (Figure 5). In the BP category, the target genes were significantly enriched in terms related to neural development and synaptic organization, including synapse organization, central nervous system neuron development, midbrain development, neuroblast division, and response to fibroblast growth factor. In the CC category, the enriched terms were predominantly associated with neuronal polarized structures and synaptic microdomains, including presynaptic active zone, presynaptic active zone cytoplasmic component, excitatory synapse, presynaptic membrane, distal axon, growth cone, cell cortex region, and organelle membrane contact site. In the MF category, transmembrane transporter binding was among the most prominent terms, together with functions related to lipid metabolism and membrane-associated regulation, including arachidonate-CoA ligase activity, long-chain fatty acid-CoA ligase activity, phosphatidylethanolamine (PE) binding, diacylglycerol (DAG) binding, guanylate cyclase activity, cyclin-dependent protein serine/threonine kinase inhibitor activity, heparin binding, fucosyltransferase activity, and oxidoreductase activity, acting on a sulfur group of donors, oxygen as acceptor.

## Discussion

TsRNAs are a class of small non-coding RNAs generated by specific cleavage of precursor or mature tRNAs encoded by either the nuclear or mitochondrial genome (Kumar et al., 2016). According to cleavage site and precursor origin, tsRNAs are broadly classified into tRNA halves (tiRNAs) and tRNA-derived fragments (tRFs). TiRNAs are typically 29–50 nt in length and are mainly produced by angiogenin (ANG)-mediated cleavage within the anticodon loop of mature tRNAs, yielding 5′-tiRNAs and 3′-tiRNAs (Li and Hu, 2012). In contrast, tRFs are shorter, usually 14–29 nt, and can originate from either precursor or mature tRNAs; although Dicer was initially implicated in their biogenesis, subsequent studies have shown that at least a subset of tRFs is generated and functions in a Dicer-independent manner (Kuscu et al., 2018). Based on their positions within the parental tRNA, tRFs can be further divided into tRF-5, tRF-3, tRF-1, tRF-2, and internal tRFs (i-tRFs) (Wang et al., 2020).

Importantly, tsRNA biogenesis is shaped not only by cleavage pattern but also by tissue context, developmental stage, and cell identity. Given the marked cellular heterogeneity of the brain and the distinct tRNA transcriptional programs across neuronal and glial populations, brain tsRNA landscapes are expected to exhibit pronounced regional and cell-type specificity (Dubnov et al., 2024; Kapur et al., 2024). Consistent with this, 5′ tRNA halves are enriched in the primate hippocampus, whereas 3′ tRNA halves predominate in the porcine hippocampus and amygdala (Haack et al., 2019; Jehn et al., 2020). These observations support the concept that tsRNA dysregulation in neurological disease is biologically meaningful rather than stochastic.

In this context, our data show a marked remodeling of the mt-tsRNA landscape in the striatum of PD model mice, indicating a broad disturbance of mitochondria-derived small RNA homeostasis under disease conditions. Notably, the observed changes were not restricted to a single mt-tsRNA species, but instead suggested disruption of a wider network governing mt-tRNA processing, cleavage, and turnover. Although the striatum is not the primary site of dopaminergic neuronal cell bodies, it is a functionally critical PD-related region with high metabolic demand and strong dependence on mitochondrial integrity. Therefore, the mt-tsRNA alterations identified here are unlikely to represent merely peripheral epiphenomena and instead may reflect central pathogenic events linked to PD-associated mitochondrial stress. This interpretation is consistent with previous reports showing aberrant tRF signatures in the substantia nigra, cerebrospinal fluid, and blood of patients with PD, including increased levels of tRFs containing a conserved RGTTCRA motif and a reduction in mitochondria-derived tRFs (Madrer et al., 2025), as well as disease-specific tsRNA profiles in the substantia nigra of parkin/POLG-deficient mice (Baindoor et al., 2024). Together, these findings support the view that tsRNA, and particularly mt-tsRNA, dysregulation is a molecular feature of PD-associated mitochondrial stress and translational imbalance.

The alteration of tsRNA levels is co-regulated by multiple factors, among which the most critical upstream determinants include nuclease activity, cellular stress states, and the homeostasis of parental tRNA chemical modifications (Akiyama and Ivanov, 2023; Kuhle et al., 2023; Suzuki, 2021). Stimuli such as oxidative stress, hypoxia, and metabolic pressure can activate ANG-mediated tRNA cleavage, thereby driving the rapid accumulation of tiRNAs and certain tRFs (Guo et al., 2023; Yamasaki et al., 2009); concurrently, processing enzymes like Dicer and RNase Z/ELAC2 are also involved in the biogenesis of specific tsRNAs (Goodarzi et al., 2015; Veneziano et al., 2016). In addition, the tRNA modification status directly determines its susceptibility to endonucleolytic cleavage. Modifications such as NSUN2-mediated *m^5^C* and queuosine can, to a certain extent, protect tRNAs from cleavage; conversely, when these modifications are absent or insufficient, tRNAs become more prone to fragmentation and generate aberrant tsRNAs (de Crécy-Lagard et al., 2019; Zhou and Jiang, 2026). Nearly half of pathogenic mtDNA mutations are clustered within the mt-tRNA region, where sequence alterations or defective modifications can readily impair tRNA stability and decoding capacity, thereby preferentially inducing the specific generation of mt-tsRNAs. Accordingly, fluctuations in mt-tsRNA expression may serve as sensitive downstream indicators of impaired mitochondrial translation and dysregulated RNA processing (Yarham et al., 2010; Zifa et al., 2007). Previous studies have shown that the MELAS-associated MT-TL1 m.3243A > G mutation results in defective mt-tRNA modification, impaired electron transport chain function, and the generation of specific mt-tRFs, which in turn feed back on the expression of nuclear-encoded mitochondrial metabolic genes and directly contribute to disease phenotypes (Dubeau et al., 2000; Fornuskova et al., 2008). Collectively, these findings suggest the presence of a close bidirectional feedback mechanism between mt-tsRNAs and mitochondrial function. On the one hand, mitochondrial dysfunction can reshape the expression profile of mt-tsRNAs; on the other hand, aberrantly expressed mt-tsRNAs may further aggravate defects in mitochondrial translation, metabolic imbalance, and cellular stress.

In the present study, we demonstrated that mimic-mediated upregulation of the target mt-tsRNA in SH-SY5Y cells effectively reduced MPP^+^ induced reactive oxygen species (ROS) accumulation and restored mitochondrial membrane potential, as assessed by JC-1 staining, indicating a critical mitochondrial protective effect of this fragment. This bidirectional regulatory network has also been corroborated by complementary evidence from aging-related studies. Mechanistic investigations have shown that aging induces Ser28 dephosphorylation of ANG and promotes its translocation to the cytoplasm, where it cleaves tRNA^Glu to generate Glu-5′tsRNA-CTC. After translocation into mitochondria, this fragment competitively inhibits the aminoacylation of mt-tRNA^Leu, thereby suppressing mitochondrial protein translation, disrupting cristae organization, and promoting ubiquitin-mediated degradation of glutaminase (GLS) in the inner mitochondrial membrane, ultimately leading to impaired glutamate biosynthesis and synaptic dysfunction (Li et al., 2024). Therefore, the functional validation of the target mt-tsRNA in the present study further supports its designation as a regulatory effector molecule rather than a passive biomarker. Nevertheless, its hierarchical role should still be interpreted with caution at the current stage, as it may represent both a reactive product of mitochondrial injury and an active participant in the amplification of Parkinson’s disease pathology through regulation of downstream pathways.

Mechanistically, tsRNAs exert diverse functions, among which their miRNA-like activity is particularly noteworthy. Previous studies have shown that certain tRFs, especially tRF-3s and tRF-5s, can associate with AGO proteins and recognize target mRNAs through seed-pairing-like interactions, thereby repressing translation or reducing transcript stability (Kumar et al., 2014). Stress conditions markedly induce tRF production; under these circumstances, defects in post-transcriptional tRNA modifications promote the generation of tRFs that can mimic miRNA-like modes of gene silencing (Skeparnias et al., 2020). Notably, mitochondria-derived tsRNAs/rsRNAs that are specifically downregulated in AD brain tissue have been proposed to target lysosomal genes via AGO2-dependent, miRNA-like mechanisms. Their deficiency may disrupt lysosomal homeostasis and impair Aβ clearance, thereby contributing to AD pathogenesis (Zhang et al., 2025). In this study, downregulated mitochondrial tsRNAs of 15–25 nt were subjected to miRDB-based target prediction analysis.

In the cellular component category, enriched terms were mainly localized to regions involved in synaptic vesicle docking, fusion, and neurotransmitter release, suggesting that altered mt-tsRNA-related networks may be linked to presynaptic dysfunction. This is notable given that synaptic impairment is increasingly considered an early event in Parkinson’s disease (PD), often preceding widespread protein aggregation, neuronal loss, and overt clinical manifestations (Li et al., 2026; Uytterhoeven et al., 2025). Consistent with this interpretation, presynaptic vesicles are essential for dopamine homeostasis, and disruption of vesicle structure or cycling can directly impair dopamine storage, trafficking, and release (Jin et al., 2024). Moreover, several PD-associated genes, including SNCA, LRRK2, and PINK1, have been implicated in the regulation of synaptic vesicle dynamics, further supporting the relevance of this pathway to PD pathophysiology. The molecular function enrichment of PE binding and DAG binding further suggests that membrane lipid interactions may contribute to the observed presynaptic signature. PE is important for synaptic membrane integrity and vesicle fusion, whereas DAG-mediated signaling regulates neurotransmitter release through activation of proteins such as PKC and downstream components of the synaptic release machinery (Asadian et al., 2025; Cooke and Kazanietz, 2022; Zhang et al., 2026).

Enrichment analysis indicates mitochondrial and metabolic dysfunction. The widespread downregulation of mt-tsRNAs may reflect abnormalities in mitochondrial tRNA processing and translation, which could subsequently impair neuronal energy supply by disrupting oxidative phosphorylation and lipid metabolism (Deng et al., 2025; Ding et al., 2023). This possibility is further supported by enrichment of terms related to organelle membrane contact sites, including mitochondria-associated membranes (MAMs), which are critical for *Ca^2+^* transfer and metabolic coupling between the endoplasmic reticulum and mitochondria (Orantos-Aguilera et al., 2026; Thi My Nhung et al., 2023). Because presynaptic neurotransmitter release is highly dependent on local mitochondrial ATP production, mitochondrial dysfunction could exacerbate synaptic deficits by limiting the energetic support required for vesicle cycling and membrane recycling.

Finally, enrichment of neuroblast division and cyclin-dependent protein serine/threonine kinase inhibitor activity raises the possibility that mt-tsRNA-associated regulatory networks may also extend to pathways involved in neural precursor proliferation and differentiation. Although these findings require further validation, taken together they suggest that the predicted targets of differentially expressed mt-tsRNAs may converge on a broader functional network involving mitochondrial homeostasis, lipid metabolism, synaptic organization, and neural circuit remodeling in PD.

However, the functions of tsRNAs extend far beyond the canonical miRNA-like mode of action. TsRNAs can exert their effects through multiple mechanisms, including interactions with RNA-binding proteins, interference with translation initiation complex assembly, regulation of ribosome biogenesis, participation in stress granule formation, modulation of histone modifications, and suppression of transposable element activity (Winek and Soreq, 2025; Zhao et al., 2024). In the nervous system in particular, ANG-induced tRNA halves may exert protective effects during acute stress by promoting stress granule assembly and restraining excessive translation (Chen et al., 2021; Ivanov et al., 2014), yet under chronic stress conditions, they may conversely become components of sustained translational repression and inflammatory amplification (Basavaraju et al., 2022; Winek et al., 2020). Therefore, the target-gene prediction and GO enrichment results obtained for the target mt-tsRNA in the present study should be regarded as evidence for potential functional directions rather than as direct proof of its molecular mechanism. Although we observed significant dysregulation of mt-tsRNAs in the PD model and performed an initial functional validation of the target molecule, it remains difficult to fully determine whether these changes represent disease-driving events, compensatory adaptive responses, or a combination of both.

In the context of neurological disorders, tsRNAs have emerged as molecules of potential biomarker relevance. A representative example, as discussed above, is the characteristic alteration pattern reported by Paldor et al. and Madrer et al. (2025), namely the upregulation of RGTTCRA motif-associated tRFs and the downregulation of mitochondria-derived tRFs. Notably, this pattern was consistently observed across the substantia nigra, cerebrospinal fluid, and peripheral blood, suggesting its potential utility not only in distinguishing patients with PD from healthy controls, but also in identifying pre-symptomatic individuals at elevated risk of developing PD. In addition, patients with AD exhibit abnormalities in both the expression profiles and abundance of small non-coding RNAs in the cerebral cortex, accompanied by characteristic alterations in RNA chemical modifications. Among these changes, 30–40 nt tsRNA fragments derived from tyrosine and arginine tRNAs were significantly decreased (Zhang et al., 2020). Specific plasma tRNA fragments have also been reported to rise several hours before clinical seizure onset in a subset of patients with chronic epilepsy, indicating their potential value as dynamic biomarkers of disease activity (Hogg et al., 2019). In amyotrophic lateral sclerosis (ALS), a 5′ tsRNA derived from valine tRNA was associated with slower disease progression, suggesting possible prognostic significance (Hogg et al., 2020). Taken together, these findings indicate that disease-associated alterations in tsRNAs are detectable not only in affected neural tissues, but also in accessible biofluids, underscoring their promise as minimally invasive biomarkers for neurological disorders.

In summary, our findings indicate that mt-tsRNA dysregulation is closely associated with mitochondrial stress in PD and is unlikely to represent a mere byproduct of RNA degradation. The broad remodeling of the mt-tsRNA landscape in the striatum of PD model mice, together with the protective effect of the selected mt-tsRNA against MPP^+^ induced mitochondrial injury in SH-SY5Y cells, supports the notion that mt-tsRNAs may function both as sensitive indicators of mitochondrial dysfunction and as active regulators of disease-related pathways. Although the precise molecular mechanisms remain to be fully defined, our bioinformatic and functional data collectively suggest that mt-tsRNAs may participate in PD pathophysiology through networks related to mitochondrial homeostasis, synaptic function, and metabolic regulation. These findings provide a basis for future studies investigating mt-tsRNAs as mechanistic contributors, candidate biomarkers, and potential therapeutic targets in PD.

## Materials and methods

### Animals and MPTP treatment

Male C57BL/6J mice (8 weeks old, 18–22 g) were obtained from Jiangsu Jicui Yaokang Biotech Co., Ltd.; certificate number: SCXK (SU) 2023-0009; license number: 2024030. Animals were housed under specific pathogen-free conditions at a controlled temperature of 20–22°C and relative humidity of 45%–65%, with a 12 h light/dark cycle and free access to food and water. All animal experiments were approved by the Institutional Animal Care and Use Committee of Shanghai Municipal Hospital of Traditional Chinese Medicine, affiliated to Shanghai University of Traditional Chinese Medicine (approval No. [2025098]) and were performed in accordance with the relevant institutional and national guidelines for laboratory animal care.

Mice were randomly allocated to a control group or an MPTP-treated group (*n* = 8 per group). To establish a subacute PD model, mice in the MPTP group received daily intraperitoneal injections of MPTP (MedChemExpress, cat.no.HY-15608) at a dose of 25 mg/kg for 10 consecutive days. Control mice received an equal volume of sterile saline following the same schedule. Behavioral tests were conducted within 48 h after the final injection, and tissue samples were subsequently collected for molecular analyses.

### Behavioral tests

All behavioral assessments were performed by investigators blinded to group allocation. Mice were acclimated to the testing room for at least 30 min before testing, and all apparatuses were cleaned with 75% ethanol or an equivalent odor-neutralizing solution between trials.

#### Open field test

The open field test was used to evaluate spontaneous locomotor activity and exploratory behavior. Each mouse was placed individually in the center of a square open-field arena (40 × 40 × 40 cm) and allowed to explore freely for 5 min. Animal movements were recorded using an automated video-tracking system (Shanghai Xinruan Information Technology Co., Ltd., VisuTrack 2.0). Total distance traveled and mean velocity in the center area were analyzed.

#### Pole test

The pole test was used to assess motor coordination and bradykinesia-related performance. A rough-surfaced vertical pole (50 cm in height and 1 cm in diameter) was used. Each mouse was placed head-upward on the top of the pole, and the T-total was recorded. Each mouse was tested three times, and the mean value was used for subsequent statistical analysis.

#### Wire hang test

The wire hang test was performed to assess forelimb grip strength and motor endurance. Each mouse was placed on a horizontally suspended metal wire (2 mm in diameter) positioned 30 cm above a padded surface and allowed to hang using its forelimbs. The latency to fall was recorded, with a cutoff time of 60 s. Each mouse was tested three times with 5 min intervals between trials, and the average value was used for analysis.

### Tissue collection

After completion of behavioral testing, mice were anesthetized with pentobarbital sodium (50 mg/kg, intraperitoneal injection) and euthanized by decapitation. Whole brains were rapidly removed, and the bilateral striata were dissected on ice according to a standard mouse brain atlas. Tissue samples were immediately snap-frozen in liquid nitrogen and stored at −80°C until use for RNA extraction and protein analysis.

### Cell culture and transfection

SH-SY5Y cells were cultured in Dulbecco’s modified Eagle’s medium/Nutrient Mixture F-12 (DMEM/F12; Servicebio, Wuhan, China) supplemented with 10% fetal bovine serum (FBS; Servicebio, Wuhan, China) and 1% penicillin–streptomycin (Servicebio, Wuhan, China) at 37°C in a humidified incubator with 5% CO_2_. Cells were seeded into 6-well plates and allowed to grow until they reached approximately 40–60% confluence before transfection.

The tsRNA mimic and corresponding negative control (mimic-NC) were synthesized by GenePharma (Shanghai, China). The sequences of mitochondrial tsRNA mimic were as follows:

**Table:** 

Sense	5′-UGGCCUACACCCAGAAGAUUUCAUGACCAAU GAACACUCUGACC-3′
Antisense	5′-UCAGAGUGUUCAUUGGUCAUGAAAUCUUC UGGGUGUAGGCCAUU-3′

Transfection was performed using Lipofectamine 3000 reagent (Invitrogen, USA) according to the manufacturer’s instructions. Briefly, SH-SY5Y cells were transfected with tsRNA mimic or mimic-NC at a concentration of 50 nM or 100 nM. After 24 h of transfection, the cells were harvested for subsequent analyses. For functional experiments, cells were collected at the indicated time points post-transfection.

### CCK-8

CCK-8 assays were performed to evaluate the cytotoxicity. Briefly, cultures were plated on the 96-well plates at the density of 2 × 10^4^ cells/well. After different treatments, the absorbance at 450 nm was measured by a microplate reader.

### RT-qPCR analysis of transfection efficiency

Total RNA was isolated from SH-SY5Y cells using TRIzol reagent (Invitrogen, USA), and its concentration and purity were determined with a NanoDrop spectrophotometer (Thermo Fisher Scientific, USA). For tsRNA quantification, 1 μg of total RNA was reverse-transcribed into cDNA using tsRNA-specific RT primers (GenePharma, Shanghai, China) in a 20 μL reaction system, according to the manufacturer’s instructions.

Quantitative real-time PCR (qPCR) was subsequently performed in a 20 μL reaction volume using SYBR Green qPCR Master Mix on a Bio-Rad real-time PCR system (Bio-Rad, USA). The relative expression level of tsRNA was normalized to U6 as the internal control and calculated using the *2^–Δ^* ^Δ^ *^Ct^* method. All reactions were performed in triplicate. The primer sequences used were as follows:

**Table:** 

mt-tsRNA	F primer	5′-TGGCCTACACCCAGAAGATTTC-3′
	R primer	5′-TATCCTTCTTCACGACTCCTTCAC-3′
U6	F primer	5′-CTCGCTTCGGCAGCACA-3′
	R primer	5′-AACGCTTCACGAATTTGCGT-3′

### Measurement of intracellular ROS and mitochondrial membrane potential

Intracellular ROS and mitochondrial membrane potential in SH-SY5Y cells were evaluated using a Reactive Oxygen Species Assay Kit and a JC-1 Mitochondrial Membrane Potential Assay Kit (Beyotime Biotechnology, Shanghai, China), respectively, according to the manufacturer’s instructions. SH-SY5Y cells were seeded into confocal dishes and subjected to the indicated treatments. For ROS detection, cells were incubated with DCFH-DA working solution at 37°C for 20–30 min in the dark and then washed three times with PBS. For MMP analysis, cells were incubated with JC-1 working solution at 37°C for 20 min in the dark and washed with JC-1 staining buffer. Fluorescence images for both ROS and MMP were immediately captured using a laser scanning confocal microscope (TCS SP8; Leica Microsystems, Wetzlar, Germany).

### TH immunohistochemistry

Paraffin sections (4 μm) were baked at 60°C for 15 min, deparaffinized in xylene, and rehydrated through graded ethanol (100%, 90%, and 80%) to distilled water, followed by equilibration in PBS. Antigen retrieval was performed by boiling the sections in 10 mM sodium citrate buffer, and the slides were then cooled to room temperature for 30 min. After washing, endogenous peroxidase activity was quenched with 3% hydrogen peroxide. Sections were then blocked at room temperature for 1 h and incubated overnight at 4°C with TH antibody (rabbit, Cell Signaling Technology, 1:1,000). After washing, sections were incubated with HRP-conjugated secondary antibody, followed by DAB development and hematoxylin counterstaining. Finally, the sections were dehydrated, cleared, and mounted.

Images were acquired under identical settings. TH immunoreactivity in the substantia nigra was quantified in ImageJ 1.54p after background subtraction. Integrated optical density and area were measured, and AOD was calculated as IOD/area. Mean values per animal were used for statistical analysis.

### Western blot analysis

Frozen striatal tissues were homogenized in ice-cold RIPA lysis buffer (Beyotime, cat.no.P0013B) supplemented with protease inhibitor cocktail (Beyotime, cat.no.ST506-2). Lysates were incubated on ice for 30 min and centrifuged at 12,000 × g for 15 min at 4°C. The supernatants were collected, and protein concentrations were determined using a BCA protein assay kit (Solarbio, cat.no.PC0020) according to the manufacturer’s instructions.

Equal amounts of protein were mixed with loading buffer, denatured at 95°C for 5 min, separated by SDS-PAGE on 10% polyacrylamide gels, and transferred onto PVDF membranes. Membranes were blocked with 5% non-fat milk in TBST for 1h at room temperature and then incubated overnight at 4°C with primary antibodies against tyrosine hydroxylase (TH; rabbit, Cell Signaling Technology, cat.no.58844, 1:1000), dopamine transporter (DAT; rabbit, Abcam, cat.no. AB184451, 1:1000), and β-actin (rabbit, Servicebio, cat.no.GB15003, 1:6000) as the loading control.

After washing with TBST, membranes were incubated with the appropriate HRP-conjugated secondary antibodies (Abmart, cat.no.M21003S, 1:10000) for 1h at room temperature. Immunoreactive bands were visualized using an enhanced chemiluminescence kit and captured with a chemiluminescence imaging system (Bio-Rad, ChemiDoc MP). Band intensities were quantified using ImageJ software (National Institutes of Health, Bethesda, MD, USA), and target protein expression levels were normalized to the loading control.

### Small RNA isolation

Total RNA was extracted from frozen striatal tissues using TRIzol reagent (Invitrogen, 15596026) according to the manufacturer’s instructions. Briefly, tissues were homogenized in TRIzol, followed by phase separation with chloroform and RNA precipitation with isopropanol. The RNA pellet was washed with 75% ethanol, dissolved in RNase-free water, and quantified using a NanoDrop 2000 spectrophotometer (Thermo Fisher Scientific, USA). RNA purity was assessed based on the absorbance ratios at 260/280 nm and 260/230 nm.

Small RNAs were enriched by electrophoretic separation on a 15% denaturing urea-polyacrylamide gel, with the procedure adapted from a previously described protocol for small RNA isolation and characterization (Shi et al., 2025). RNA fragments of 15–50 nt were identified using a small RNA size marker (Beyotime, cat.no.R0207), excised from the gel, and eluted overnight at 4°C in 0.3 M sodium acetate. The eluate was centrifuged at 12,000 × g for 10 min at 4°C, and the supernatant was subjected to ethanol precipitation by adding 3 volumes of ethanol, 1/10 volume of 3 M sodium acetate, and linear acrylamide as carrier. After incubation at −20°C for 2 h, the samples were centrifuged at 12,000 × g for 25 min. The resulting RNA pellet was resuspended in nuclease-free water, quantified, and stored at −80°C until library preparation or further analysis.

### PANDORA-seq library preparation and sequencing

PANDORA-seq was performed essentially as previously described (Shi et al., 2025). In brief, purified 15–50 nt RNAs were subjected to enzymatic pretreatment to improve the recovery of modified small RNAs. Sequencing libraries were prepared using the NEBNext Small RNA Library Prep Kit (New England Biolabs, cat.no.E7560S) according to the manufacturer’s instructions. Briefly, adapters were ligated to both ends of the processed RNA, followed by reverse transcription and PCR amplification. Library fragments of 130–170 bp were size-selected by PAGE, recovered, and quantified using a Qubit fluorometer (Thermo Fisher Scientific, USA). Library quality was further assessed using an Agilent 2100 Bioanalyzer (Agilent Technologies, USA). Qualified libraries were sequenced on an Illumina platform (NovaSeq 6000, Illumina) in single-end 50 bp mode.

### Bioinformatic analysis

Raw sequencing reads were processed using Cutadapt 1.11 to remove adapter sequences and low-quality reads. Reads ranging from 15 to 45 nt in length after trimming were retained for downstream analysis. The cleaned reads were annotated using SPORTS 1.1 against reference databases to classify small RNA species, including miRNAs, tsRNAs, rsRNAs, snRNAs, and related subclasses.

Differential expression analysis was performed using the edgeR package4.10.0 in R 4.2.2 Read counts were normalized using the trimmed mean of M-values (TMM) method. Differentially expressed small RNAs between the control and MPTP groups were identified using the likelihood ratio test. Small RNAs with a false discovery rate (FDR) < 0.05 and | log2 fold change| > 1 were considered significantly differentially expressed. PCoA and volcano plots were generated using the FactoMineR and ggplot2 packages in R, respectively.

### Target prediction and GO enrichment analysis

Putative target genes of differentially expressed mt-tsRNAs (15–25 nt) were predicted using miRDB^1^. Functional enrichment analysis of the predicted target genes was performed using clusterProfiler package4.20.0 or an equivalent online platform. GO analysis was conducted in the categories of BP, CC, and MF. GO terms with adjusted *P* < 0.05 were considered significantly enriched.

### Statistical analysis

Statistical analyses were performed using GraphPad Prism 8.0 and R (version 4.2.2). Data are expressed as mean ± SEM. Normality was assessed using the Shapiro–Wilk test. Differences between two groups were analyzed using an unpaired two-tailed Student’s *t*-test or Mann–Whitney U test, as appropriate. Multiple-group comparisons were performed using one-way ANOVA. For sequencing data, differential expression analysis was conducted using edgeR after TMM normalization, with Benjamini–Hochberg correction for multiple testing. Small RNAs with FDR < 0.05 and | log2 fold change| > 1 were considered significantly differentially expressed. A two-sided *P* < 0.05 was considered statistically significant.

## Data Availability

The sequencing data have been deposited in the Genome Sequence Archive (GSA) under accession number CRA045273: https://ngdc.cncb.ac.cn/gsa/search?searchTerm=CRA045273.

## Footnotes

1
https://mirdb.org/custom.html
